# Improved sensitivity of computed tomography towards iodine and gold nanoparticle contrast agents via iterative reconstruction methods

**DOI:** 10.1038/srep26177

**Published:** 2016-05-17

**Authors:** Ally Leigh Bernstein, Amar Dhanantwari, Martina Jurcova, Rabee Cheheltani, Pratap Chandra Naha, Thomas Ivanc, Efrat Shefer, David Peter Cormode

**Affiliations:** 1 Department of Bioengineering, University of Pennsylvania, Philadelphia, PA, USA; 2 Philips Healthcare, Highland Heights, OH, USA; 3 Department of Radiology, University of Pennsylvania, 3400 Spruce St, 1 Silverstein, Philadelphia, PA 19104, USA; 4 Division of Cardiovascular Medicine, University of Pennsylvania, Philadelphia, PA, USA

## Abstract

Computed tomography is a widely used medical imaging technique that has high spatial and temporal resolution. Its weakness is its low sensitivity towards contrast media. Iterative reconstruction techniques (ITER) have recently become available, which provide reduced image noise compared with traditional filtered back-projection methods (FBP), which may allow the sensitivity of CT to be improved, however this effect has not been studied in detail. We scanned phantoms containing either an iodine contrast agent or gold nanoparticles. We used a range of tube voltages and currents. We performed reconstruction with FBP, ITER and a novel, iterative, modal-based reconstruction (IMR) algorithm. We found that noise decreased in an algorithm dependent manner (FBP > ITER > IMR) for every scan and that no differences were observed in attenuation rates of the agents. The contrast to noise ratio (CNR) of iodine was highest at 80 kV, whilst the CNR for gold was highest at 140 kV. The CNR of IMR images was almost tenfold higher than that of FBP images. Similar trends were found in dual energy images formed using these algorithms. In conclusion, IMR-based reconstruction techniques will allow contrast agents to be detected with greater sensitivity, and may allow lower contrast agent doses to be used.

Computed tomography (CT) is a widely used, whole body, medical imaging technique that has high spatial and temporal resolution. Over 70 million CT scans are performed per year in the USA alone^1^, with iodinated contrast agents used in roughly half of those studies^2^. CT is used to image a wide range of anatomical structures, such as the cardiovascular system, the gastrointestinal system, the brain, joints, areas of trauma and others^3^. CT is particularly valuable for imaging the coronary arteries, for which its high speed and high spatial resolution make it arguably the best imaging approach^4^,^5^. The notable disadvantage of CT compared with other imaging technologies is the lack of sensitivity towards contrast agents. Nuclear techniques have sensitivities of around 10^−10 ^M, while magnetic resonance imaging affords sensitivities of approximately 10^−5 ^M^6^. The sensitivity of CT towards contrast agents is about 10^−2 ^M^7^. The consequence for CT is that large doses of contrast agents are needed.

Intravenous contrast agents FDA-approved for CT are iodinated small molecules such as iopamidol or iodixanol^8^. These iodinated agents have a number of drawbacks including patient allergic reactions and contra-indication for use with renal insufficiency^9^, as their use in such patients may lead to acute kidney injury, an event known as contrast-induced nephropathy^10^,^11^. The latter issue is likely to increase in the coming years, as renal insufficiency is frequently caused by diabetes, which is projected to increase from 10% currently to 20–30% by 2050^12^. In addition, the half-lives of iodinated agents are very short, necessitating high doses, swift post-injection imaging and they do not preferentially accumulate in diseased sites, compared with normal tissue. Over the past decade there has been considerable interest in developing alternative agents, which has primarily focused on nanoparticle-based formulations^13^,^14^. Nanoparticles made of heavy elements such as gold^15^,^16^, bismuth^17^,^18^,^19^ or platinum^20^ attenuate X-rays more strongly than iodine^7^,^21^. Nanoparticles have been shown to be effective as long circulating contrast agents for CT^18^,^22^. In addition, nanoparticles can be used as targeted CT contrast agents^15^,^23^,^24^,^25^,^26^,^27^. Both the continued widespread use of iodinated agents and the introduction of novel, nanoparticle-based CT contrast agents would benefit from improvements in the sensitivity of CT imaging.

The reconstruction of CT images has typically been done using filtered back-projection algorithms since the introduction of CT^28^. As computing power has increased, the time for image reconstruction has decreased from hours to seconds, even as the volume of data has increased due to increased spatial resolution^29^. Hybrid iterative reconstruction methods that employ statistical models to achieve noise reduction have been introduced in the last decade, and these iterative reconstruction methods are now frequently available on modern CT scanners. More advanced model based iterative reconstruction methods have been proposed for many years, but were not practical until recent years due to the larger computing power required for this approach. One such model-based reconstruction method has been released, and requires an additional computational processing unit^30^. The main attraction of iterative reconstruction methods and model based iterative reconstruction methods versus filtered back-projection is that they typically result in lower image noise for given acquisition parameters. Most investigators have studied the ability of iterative reconstruction methods or model based iterative reconstruction methods to produce images of equivalent quantitative or diagnostic quality to filtered back-projection derived images, while using lower radiation doses^31^,^32^,^33^. However, as sensitivity can be measured in contrast to noise ratio (CNR), reduced image noise should improve the sensitivity of CT towards contrast agents. There have been a couple of reports that indicate that novel reconstruction methods can facilitate the use of lowered contrast agent doses, however a systematic study has not yet been performed^34^,^35^. We therefore sought to quantify the effect of filtered back-projection, iterative reconstruction and model based iterative reconstruction algorithms on contrast agent attenuation, image noise and sensitivity of agent detection.

We constructed two identical phantoms containing a physiologically relevant concentration range of either an FDA-approved contrast agent (iopamidol) or nanoparticles (we chose gold nanoparticles as they are frequently used in reports of novel preclinical CT contrast agents). The phantoms are schematically depicted in Supplementary Figure 1. We scanned the phantoms using a 256-slice CT scanner using three different X-ray tube voltages (80, 120 and 140 kV) and a wide range of currents (10–450 mA). The data was reconstructed using filtered back-projection, iterative reconstruction and model based iterative reconstruction algorithms, which we termed FBP, ITER or IMR respectively, resulting in a total of 174 datasets. A typical image is shown in Supplementary Figure 1. The images were analyzed for contrast agent attenuation and noise. The linearity of contrast agent attenuation with concentration, attenuation rate, noise and CNR were studied. Dual energy ratiometric maps were formed from the different voltages datasets. The noise and sensitivity of detection for contrast agents was determined for this dual energy imaging approach also. This approach is summarized in Supplementary Figure 1.

## Results

### Effects of acquisition parameters and reconstruction algorithms on contrast agent attenuation

**Figure 1:**
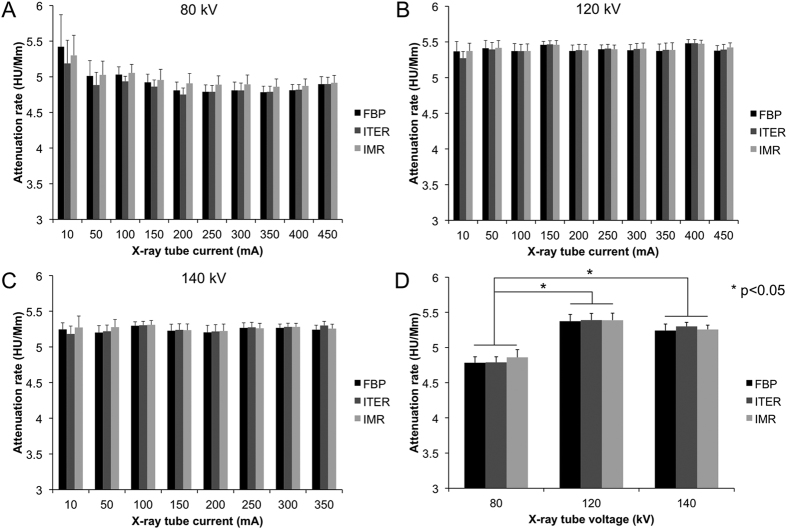
Effect of x-ray tube current and reconstruction algorithm on the attenuation rate of gold nanoparticles. **(A–C**) Data from scans performed at 80, 120 and 140 kV, respectively. (**D**) Comparison of the attenuation rate of gold nanoparticles at different X-ray tube voltages when scans are performed at 350 mA.

Prior to analyzing the effects of reconstruction algorithm on sensitivity, we examined whether the reconstruction algorithm had an effect on attenuation. The attenuation rate for the two contrast agents was determined for each set of image acquisition conditions (i.e. each voltage and current) and for each reconstruction algorithm used. As expected, we found that the attenuation was linearly correlated to concentration for both gold nanoparticles and iopamidol for each reconstruction algorithm explored, as evidenced by high R^2^ values in each case (Supplementary Figures 2 and 3). The contrast generated for each condition and algorithm for gold nanoparticles is shown in Fig. 1. As can be seen, the attenuation produced for each current was very similar for a given voltage. In the case of 80 kV, there is a slight increase in attenuation rate at low current (10 mA), but this difference was not found to be statistically significant when compared to the attenuation rate at 350 mA for any reconstruction method. This lack of significance is due to the relatively high standard error for the 10 mA value, which is likely due to few photons being received by the detector at such a low current, resulting in increased artifacts, as has been previously noted by others^36^. When the attenuation values themselves were examined (Supplementary Figure 4), it was found that the values for many of the concentrations were markedly different at 10 mA versus higher currents, indicating that scanning at such low currents is unreliable (the attenuation of the water did not substantially differ). Overall, the results from these experiments are encouraging, as they indicate that tube currents (and hence radiation dose) can be decreased and novel reconstruction methods can be used while maintaining the same contrast agent attenuation. Similar results were found for iopamidol, as can be seen in Supplementary Figure 5.

On the other hand, when the X-ray tube voltage was increased from 80 kV to higher values, the attenuation of the agents changed significantly. Figure 1D illustrates this change for gold nanoparticles at 350 mA. For gold, the attenuation rate was 4.8 HU/mM at 80 kV, 5.4 HU/mM at 120 kV and 5.3 HU/mM at 140 kV. For iodine, the attenuation rate was 3.6 HU/mM at 80 kV, 2.2 HU/mM at 120 kV and 1.8 HU/mM at 140 kV (see Supplementary Figure 5). These results are consistent with those found in previous studies^7^,^37^ and are due to the energies of the respective k-edges of gold and iodine. The k-edge of iodine is at 33.2 keV, so the attenuation of iodine becomes lower as the tube voltage increases and the energy distribution of the beam shifts to higher energies. The k-edge of gold is at 80.7 keV, therefore gold attenuates more at tube voltages over 80 kV than at 80 kV or less.

### Effect of reconstruction algorithm on image noise and CNR rate

**Figure 2:**
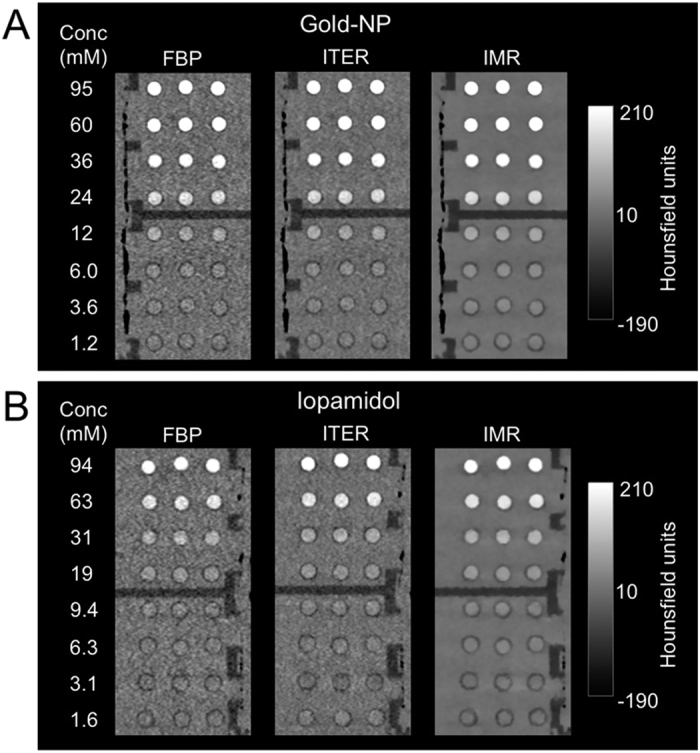
CT images of phantoms. (**A**) The gold nanoparticle (gold-NP) phantom and (**B**) the iopamidol phantom acquired at 140 kV and 350 mA reconstructed with different algorithms.

**Figure 3:**
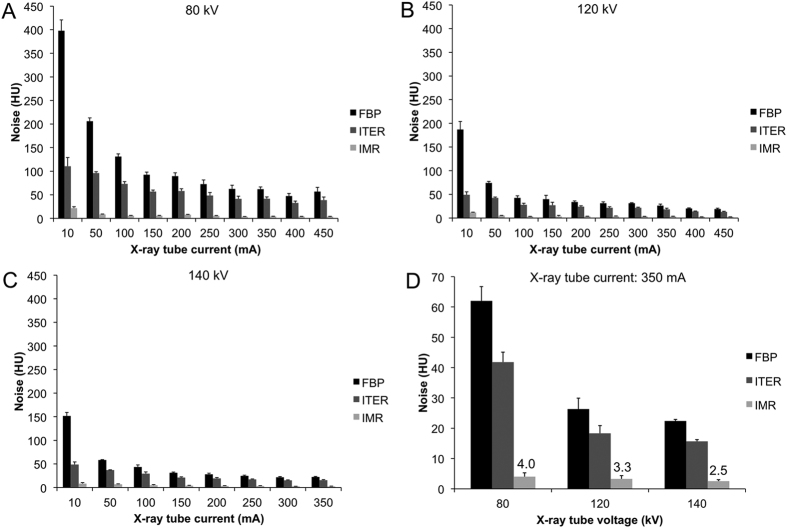
Effect of x-ray tube current, voltage and reconstruction algorithm on image noise. (**A–C**) Data from scans performed at 80, 120 and 140 kV, respectively. (**D**) Comparison of the noise of scans acquired at different X-ray tube voltages when scans are performed at 350 mA.

We found that noise is reduced when images are reconstructed with ITER or IMR as compared to FBP, as can be seen by visual inspection in Fig. 2. For all voltages and reconstruction algorithms the noise decreased with increasing current, as can be seen in Fig. 3A–C. For any given current and voltage setting, the noise was highest with FBP, lower with ITER and lowest with IMR. The reduction in noise compared to FBP varied depending on the voltage, being highest for 80 kV and decreasing as the voltage increased to 120 and 140 kV. The noise reduction was highest at low currents and became constant at higher currents. For example, the reduction in noise for ITER vs FBP was 72% at 80 kV and 10 mA, whereas it was 30–35% at 80 kV and 200–450 mA. Similarly there was a noise reduction of 94% for IMR versus FBP at 120 kV and 10 mA, whereas the noise reduction was 87–90% at 120 kV and currents of 200–450 mA. Notably, the highest noise in IMR images for a given voltage (i.e. at 10 mA) was still *less than* the lowest noise produced using FBP or ITER (i.e. at >350 mA). When FBP images acquired at 350 mA are compared visually with IMR images acquired at 10–100 mA (all acquired using 140 kV), IMR images of 50 mA are of equivalent quality, as can be seen in Supplementary Figure 6. Although the noise is quantitatively lower in the 10 mA IMR image, the lower concentration tubes cannot be clearly visually distinguished from the background. As would be expected, for a given X-ray tube current, as the voltage increases, the noise decreases, as can be seen in Fig. 3D.

**Figure 4:**
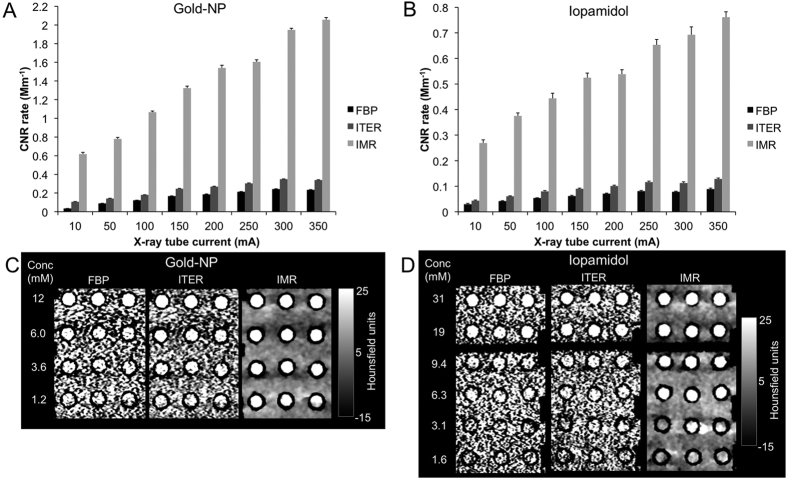
Effect of x-ray tube current and reconstruction algorithm on CNR rate at 140 kV. (**A**) Data for gold nanoparticles. (**B**) Data for iopamidol. CT images of the (**C**) gold nanoparticle phantom and (**D**) iopamidol phantom reconstructed with different algorithms when scanned at 140 kV and 350 mA displayed with a narrow Hounsfield unit window.

When the noise in images decreases, the CNR increases, as is illustrated in Fig. 4A,B. The approximately 90% reduction in noise in IMR-reconstructed images compared with FBP-reconstructed images yields a ten-fold increase in CNR rate. This effect is further illustrated in Fig. 4C,D, where, in tightly windowed images, it can be seen that 1.2 mM concentrations of gold nanoparticles can be detected when IMR is used to reconstruct the images, whereas 12 mM is the lowest concentration that can be clearly distinguished from the background in the case of FBP or ITER. In the case of iopamidol, the lowest concentration tested that could be distinguished from the background in IMR images is 6.3 mM, due to the lower attenuation rate of iodine at this X-ray tube voltage (140 kV). However, again, the lowest detectable concentration tested for FBP or ITER reconstructed images is 31 mM, a much higher concentration. This indicates that an effect of IMR reconstruction methods is to increase the sensitivity of CT towards contrast agents. For gold nanoparticles, scanning at 140 kV, for a given current, gives the highest CNR rate, irrespective of reconstruction algorithm (Supplementary Figure 7). This is because the attenuation rate for gold is greater at both 120 and 140 kV than at 80 kV (Fig. 1D), while noise is lower the higher the voltage (Fig. 3). On the other hand, the contrast to noise ratio for iopamidol is highest at 80 kV when IMR is used (Supplementary Figure 7), since the attenuation rate for iopamidol is higher at 80 kV than at 120 or 140 kV (Supplementary Figure 5). Conversely, for FBP and ITER, the iopamidol CNR is highest at 120 or 140 kV (there was not a significant difference). These results occur because the noise for IMR is very low at 80 kV compared with FBP or ITER and hence the CNR is high at this voltage (Fig. 3D). Increasing the voltage resulted in large reductions of noise for FBP and ITER, but only small reductions for IMR. Therefore the CNR for iopamidol is low at 80 kV for FBP and ITER due to their relatively high noise at this voltage.

### Effect of reconstruction algorithm on dual energy CT imaging

**Figure 5:**
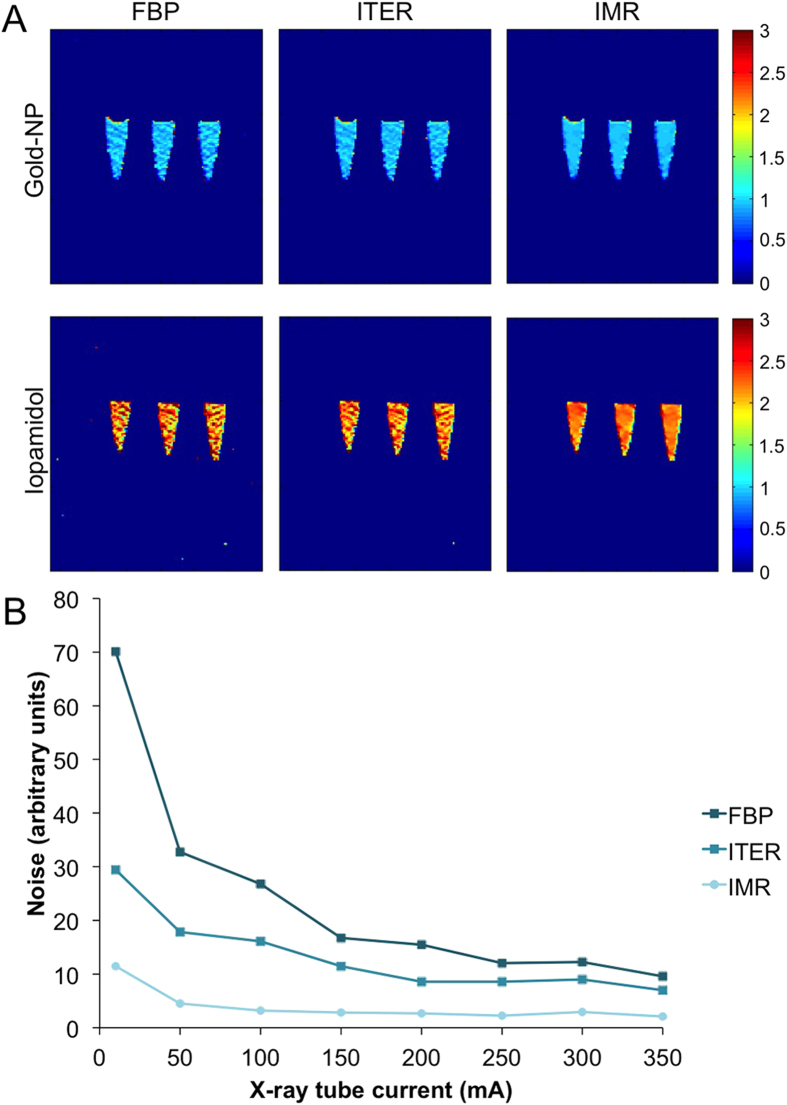
Effect of reconstruction algorithm in dual energy CT. **(A**) Dual energy CT ratiometric maps formed from 80 and 140 kV images acquired at 350 mA using different reconstruction protocols. The color bar represents the attenuation at 140 kV divided by the attenuation at 80 kV (with both attenuations adjusted by 1080 HU). (**B**) Effect of X-ray tube current and reconstruction algorithm on noise in dual energy CT ratiometric maps in 95 mM samples.

Dual energy imaging is becoming more common in CT clinical practice and is used to segment the location of contrast agents and to create virtual non-contrast images. We examined the effect of reconstruction method on dual energy CT imaging by forming ratiometric images from voltage pairs for a given current and analyzing the noise in the resulting images. We found that the pairing of 80 and 140 kV gave the best separation between gold and iodine, so we focused our analysis on this pairing. As can be appreciated visually in Fig. 5A, use of the IMR reconstruction algorithm reduced noise compared with FBP or ITER. This was confirmed by image analysis, as displayed in Fig. 5B. We found that there had been a slight positional shift for the iopamidol phantom during image acquisition, as indicated by the red rim around the samples, therefore our quantitative analysis focused on the images from the gold nanoparticle phantom. As would be expected, noise increased as current decreased (Fig. 5B), although substantial increases in noise within the samples only occurred below 150 mA. Similarly to single energy images, the noise reduction from the iterative-based reconstruction algorithms was found to be highest at low currents. The noise in images reconstructed with ITER was reduced by 45% at 50 mA compared with FBP, whereas the noise was reduced by about 27% at 350 mA. The noise reduction for IMR compared to FBP was 86% at 50 mA, but 78% at 350 mA. When examining the images visually, the consequence of this seems to be that when IMR is used, contrast agents can be accurately segmented when using currents as low as 50 mA, whereas for ITER reconstructed images the minimal current would be 150 mA and for FBP reconstructed images, 250 mA would be the lowest current that could be used (Supplementary Figure 8). When examining the images for each reconstruction algorithm and different concentrations of contrast agent, we found that IMR could specifically detect concentrations of gold nanoparticles of 24 mM or higher, whereas FBP and ITER were less sensitive being specific for concentrations of 95 and 60 mM, respectively (Supplementary Figure 9).

## Discussion

Via our experiments, we have shown that contrast agent attenuation rates are unaffected by the use of novel, iterative reconstruction algorithms, as expected. We have shown that attenuation values are only affected at the lowest X-ray tube currents. Attenuation is directly proportional to contrast agent concentration in all cases. As expected^7^, the attenuation varied with X-ray tube voltage in a manner that depends on the k-edge of the agent. We found that, depending on the data acquisition conditions, the iterative reconstruction algorithms reduced image noise by 30–94%, compared with FBP. IMR reduced noise substantially more than ITER. This leads to a consequential increase in CNR for the contrast agents examined, with sensitivity limits reduced by as much as a factor of ten. *In vivo* confirmation of the noise reduction potential of IMR has recently been published^38^. Similar effects were observed in dual energy imaging, with a reduction in image noise from iterative-based reconstruction of 27–86% compared with FBP and sensitivity improved by as much as a factor of four.

As discussed above, the currently available iodinated contrast agents suffer from limitations such as adverse, allergic reactions and being contraindicated for use with patients that have renal insufficiency. The latter issue is only expected to increase due to factors such as a rising proportion of the population that has diabetes^12^. The implications of this study are that novel, iterative model-based reconstruction techniques may allow lower doses of these iodinated contrast agents to be used, thereby potentially reducing risks or harm to patients. Molecular imaging is starting to emerge for CT^13^,^14^,^27^, especially with nanoparticle-based contrast agents, but a notable limitation is the sensitivity of CT. The results obtained herein suggest that molecular imaging with CT, when combined with iterative reconstruction methods, may be more feasible than previously thought, due to increases in sensitivity of an order of magnitude. Most studies of the effects of iterative reconstruction methods have focused on reducing radiation dose. For contrast-enhanced studies it may be necessary to make compromises between reductions in contrast agent dose and reductions in radiation dose in order to maintain CNR in the blood vessels, i.e. for some patients, such as those with impaired kidney function, the ability to reduce contrast agent dose due to maintaining the radiation dose may be a worthwhile tradeoff of risks.

The IMR algorithm is currently available on certain clinical CT scanners, although it has the limitation that an upgrade of the computing equipment is needed to perform reconstructions with this algorithm. The algorithm can be ported to any type of CT system, but the models of any new platform will need to be defined in the algorithm. IMR operates as an optimization process and included system models as well as statistical models in the optimization. System models include focal spot characteristics, detector characteristics and system geometry. Therefore, for preclinical systems, specific adaptations of the algorithm would be needed for individual scanners.

There are a number of limitations to our study. It is important to note that the results obtained in this study were derived from scanning a relatively simple phantom. The results might be somewhat different in patients due to effects of different tissues, calcified structures, possible movement, air pockets, etc. Clinical experiments will be needed to confirm the results found herein. In addition, the dual energy experiments were not carried out with a scanner specifically designed as a dual energy imaging system. Other scanners have different configurations, such as two X-ray tubes acquiring data simultaneously or dual layer detectors, so the specific results found with such scanners might vary. Some approaches to dual energy image formation involve combining the two datasets prior to reconstruction, which may have advantages over the currently used approach. Last, the most effective reconstruction method we tested (IMR) requires about three minutes to produce images on an additional computer processing unit. However, with future improvements in computational power this limitation will likely diminish.

### Summary

Our findings indicate that iterative reconstruction techniques may prove highly valuable for improved contrast agent detection. The accuracy of contrast agent detection and linearity of attenuation with agent concentration is unaffected. However the noise in CT images reconstructed with iterative algorithms can be ten-fold lower than when using filtered back-projection reconstruction. As a consequence, the CNR rate is ten-fold higher. This increase in CNR rate indicates that contrast agents can be detected with greater sensitivity when using IMR, implying that lower doses of contrast agents could be used or improving the prospects for CT as a molecular imaging technique. The effects of IMR for reducing noise and improving sensitivity also apply in dual energy imaging techniques. Further *in vivo* or clinical investigations are warranted, but it seems likely that novel iterative reconstruction methods will have a notable impact on CT contrast agent use and development.

## Materials and Methods

### Gold nanoparticle synthesis

We used an easily synthesized gold nanoparticle formulation in this study as a model CT contrast agent. 3.6 nm gold nanoparticles were used in this study that were synthesized via previously reported methodology^7^,^39^. In short, hydrophobically coated gold nanocrystals that were synthesized by the method of Brust^40^ were subsequently coated with myristoyl hydroxy phosphatidylcholine (MHPC). The gold nanocrystals and MHPC (1:1 weight ratio) were dissolved in a 9:1 chloroform:methanol solvent mixture. This solution was dripped slowly into hot (80 °C) deionized water. Large aggregates were removed by low-speed centrifugation for a brief period and retention of the supernatant. Excess phospholipids were removed via ultra-centrifugation for an hour on KBr solution (d = 1.25 g/ml) and discarding the clear upper layer. The resulting solution was transferred to phosphate buffered saline (PBS) and concentrated via use of molecular weight cut-off concentrator tubes. Gold nanoparticle concentration was determined via inductively coupled plasma mass spectrometry (ICP-MS). Concentrations are given as the number of gold atoms. 3.6 nm gold nanoparticles contain 1420 gold atoms, on average. Therefore the highest concentration used in this study, 95 mM of gold atoms, corresponds to 67 μM on a nanoparticle basis.

### Phantom construction

The two phantoms used were formed in a similar fashion. In the first gold nanoparticles were used; in the second iopamidol was used. Gold nanoparticles were diluted with PBS to 1.2, 3.6, 6.0, 12, 24, 36, 60 and 95 mM. 1.5 ml of each solution was pipetted into 3 × 1.5 ml centrifuge tubes. The tubes were sealed with vacuum grease and wrapped in parafilm to prevent water entry. The concentrations of the iopamidol samples were 1.6, 3.1, 6.3, 9.4, 19, 31, 63 and 94 mM. The tubes were placed in a four-way rack and secured with parafilm. A custom made plastic container was used that was 24 cm in width. An empty rack was placed in the container and the rack with samples placed on it in order to raise the samples to the center of the phantom. The racks were taped to the bottom of the container and water was added to a height of 21 cm, in order to mimic the conditions in a human abdomen. A schematic depiction of the phantom is displayed in Supplementary Figure 1.

### Phantom imaging

The phantoms were scanned using a 256-slice Brilliance iCT scanner (Philips Healthcare, Cleveland, Ohio, USA). The following parameters were held constant for each scan: collimation 128 × 0.625 mm, field of view (FOV) 350 mm, matrix 512 × 512, slice thickness of 1 mm and increment of 0.5 mm. The phantoms were scanned at 80, 120 and 140 kV. Scans were performed at 10, 50, 100, 150, 200, 250, 300 and 350 mA for each X-ray tube voltage. Scans were also performed at 400 and 450 mA for 80 and 120 kV. These currents were not available on the scanner for 140 kV. The raw data was obtained from the scanner and reconstructed off-site using either filtered back-projection (Standard), iDose (Level 4) or IMR algorithms, referred to as FBP, ITER and IMR respectively (the algorithms are directly comparable since they were all sourced from Philips Healthcare, Cleveland, Ohio, USA). This created a total of 174 sets of images. A schematic depiction of the data acquisition and analysis process is given in Supplementary Figure 1.

### Image analysis

Images were analyzed using Osirix 64 bit (v3.7.1, Pixmeo, Geneva, Switzerland). A circular region of interest (ROI) was placed in each tube and the average Hounsfield unit value was recorded in each ROI. Three slices were analyzed per set of images. The attenuation in each tube was averaged over the three slices and then for each contrast agent concentration (since there were three samples per concentration). The attenuation and standard deviation in the water adjacent to the samples was recorded for each slice analyzed. In this study, noise was defined as the standard deviation of the pixels in the water ROI. From these values the attenuation and CNR rates were calculated. Attenuation rate has been previously defined as attenuation per unit concentration (given in HU/mM)^7^,^41^. The LINEST function in Excel (Microsoft) was used to determine the attenuation rate (m_x_) and standard error of fit (e_x_) for each phantom and image set. This allowed us to calculate t-values when comparing results from different image sets via the following equation:


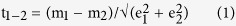


T-values were compared to the 5% reference value, which is 2.14 when using fourteen degrees of freedom, to determine significant differences. CNR was defined as:


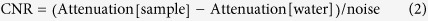


The CNR was determined for each sample and the CNR rate (CNR per unit concentration, units of mM^−1^) was calculated and statistical comparisons were performed in the same way as for the attenuation rate.

To analyze the effects of current/reconstruction method on dual energy discrimination between contrast agents, we made ratiometric image maps using Matlab between each voltage for each current, reconstruction method and phantom. The code used is given in the Supplemental Digital Content. A 1080 HU threshold was applied to each image prior to creating ratiometric images in order to reduce signal from water. The noise in the water and samples were determined from the ratiometric images via Matlab-based image analysis.

### Supplemental digital content listing

A supplemental digital content file has been submitted that contains plots of attenuation versus concentration, plots of attenuation rate at different X-ray tube voltages and currents for iopamidol, CT images of the phantoms acquired under different conditions, contrast to noise ratios at different X-ray tube voltages, ratiometric images and the Matlab code used to form the ratiometric maps.

## Additional Information

**How to cite this article**: Bernstein, A. L. *et al*. Improved sensitivity of computed tomography towards iodine and gold nanoparticle contrast agents via iterative reconstruction methods. *Sci. Rep.* **6**, 26177; doi: 10.1038/srep26177 (2016).

## Supplementary Material

Supplementary Information
