# Supplementary material for: Improved sensitivity of computed tomography towards iodine and gold nanoparticle contrast agents via iterative reconstruction methods
Source: Sci Rep. 2016 May 17;6:26177. doi: 10.1038/srep26177 (PMC4868985; doi:10.1038/srep26177)
Supplement: Supplementary Information [file srep26177-s1.pdf]

# Improved sensitivity of computed tomography towards iodine and gold nanoparticle contrast agents via iterative reconstruction methods


Ally Leigh Bernstein,[1] Amar Dhanantwari,[2] Martina Jurcova,[3] Rabee Cheheltani,[3] Pratap Chandra Naha,[3] Thomas Ivanc,[2] Efrat Shefer,[2] David Peter Cormode,[1,3,4]*

1) Department of Bioengineering, University of Pennsylvania, Philadelphia, PA, USA

2) Philips Healthcare, Highland Heights, OH, USA

3) Department of Radiology, University of Pennsylvania, 3400 Spruce St, 1 Silverstein, Philadelphia, PA 19104, USA, Tel: 215-615-4656, Fax: 215-662-3037 david.cormode@uphs.upenn.edu

4) Division of Cardiovascular Medicine, University of Pennsylvania, Philadelphia, PA, USA

* Corresponding author

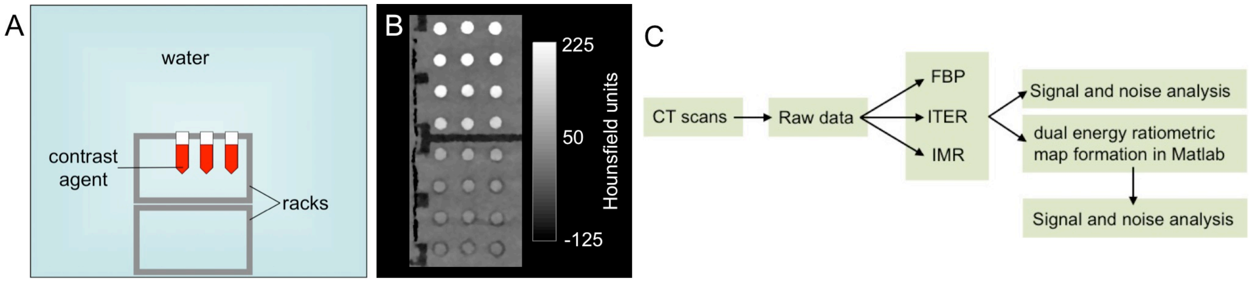


**Supplementary Figure 1 A.** Schematic depiction of phantom design. **B.** Coronal projection of the gold nanoparticle phantom acquired at 100 mA and 140 kV. **C.** Schematic depiction of data acquisition and analysis.

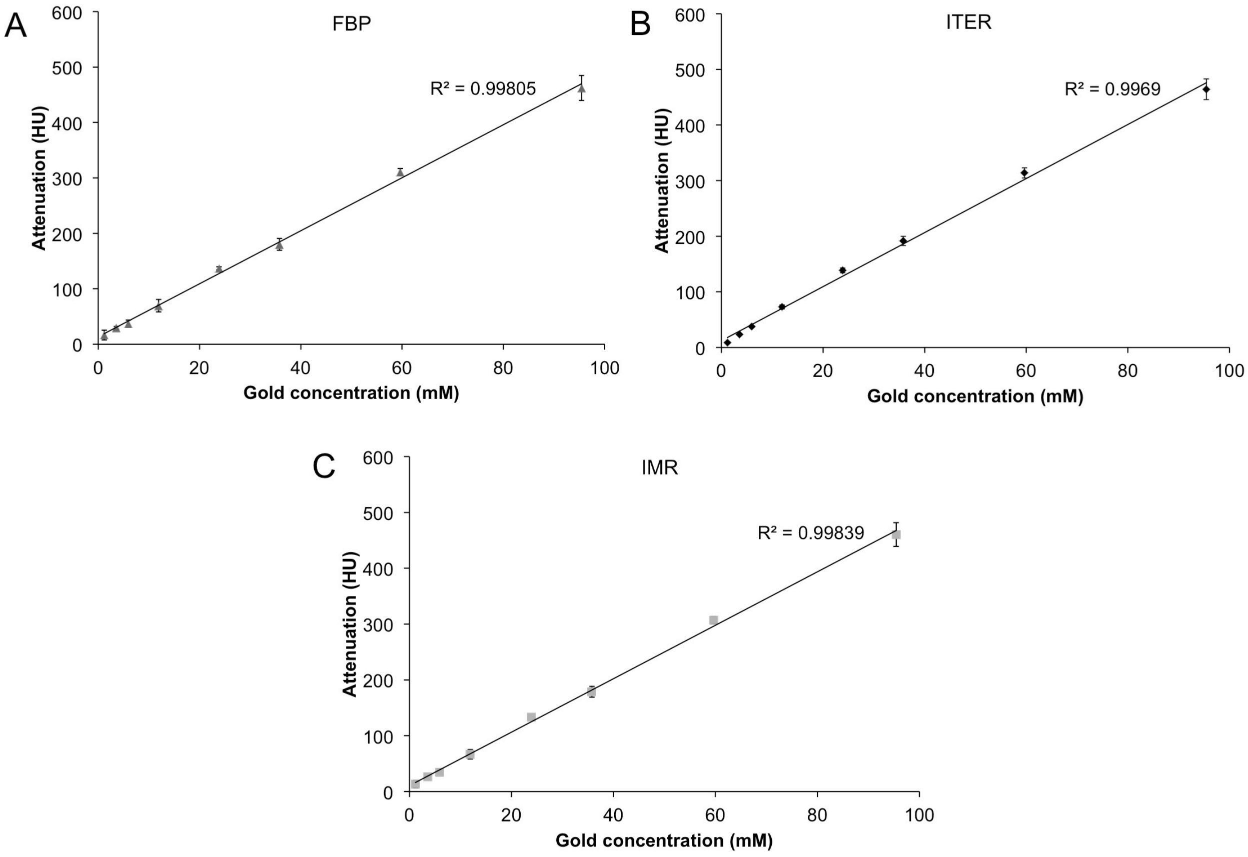

**Supplementary Figure 2** Attenuation versus concentration for gold nanoparticles in images acquired at 80 kV and 350 mA and reconstructed with **A.** FBP, **B.** ITER and **C.** IMR algorithms. Error bars are one standard deviation.

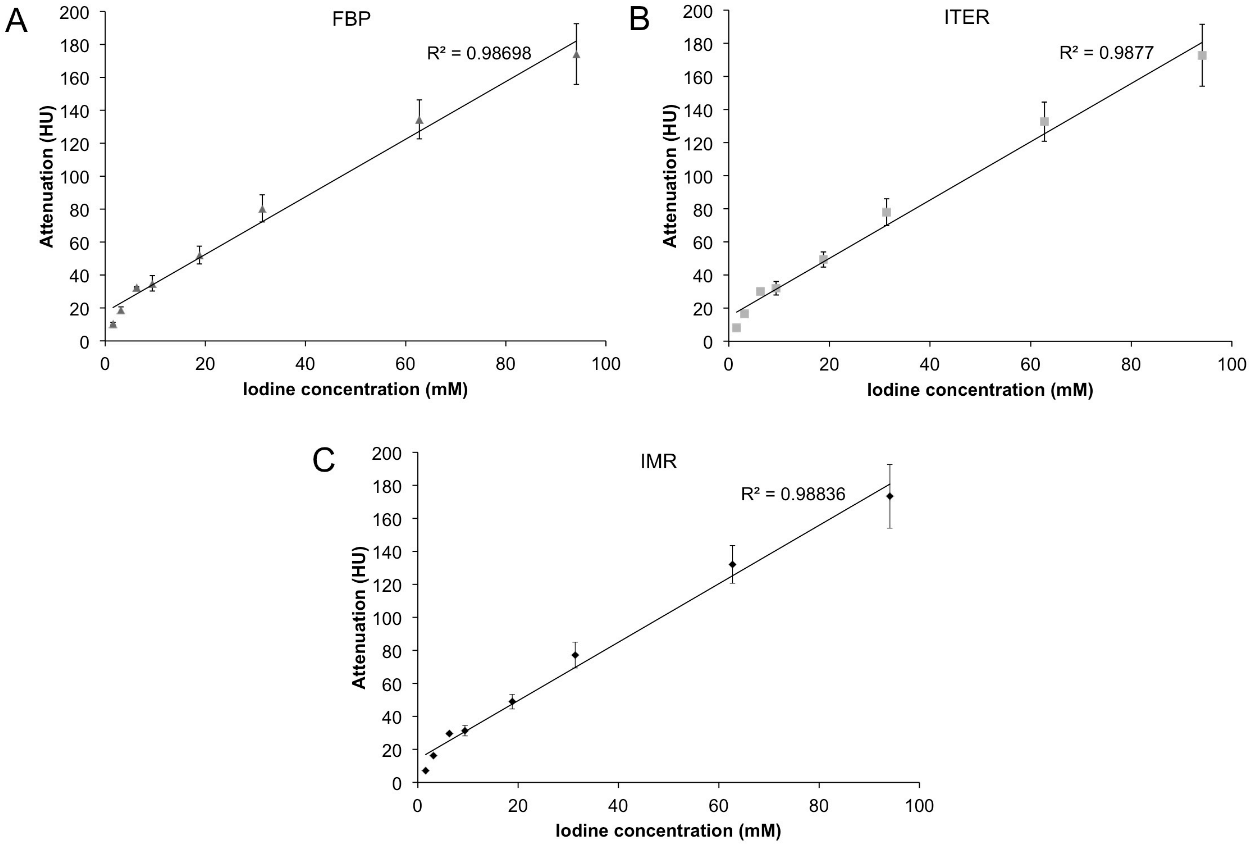


**Supplementary Figure 3** Attenuation versus concentration for iopamidol in images acquired at 140 kV and 350 mA and reconstructed with **A.** FBP, **B.** ITER and **C.** IMR algorithms. Error bars are one standard deviation.

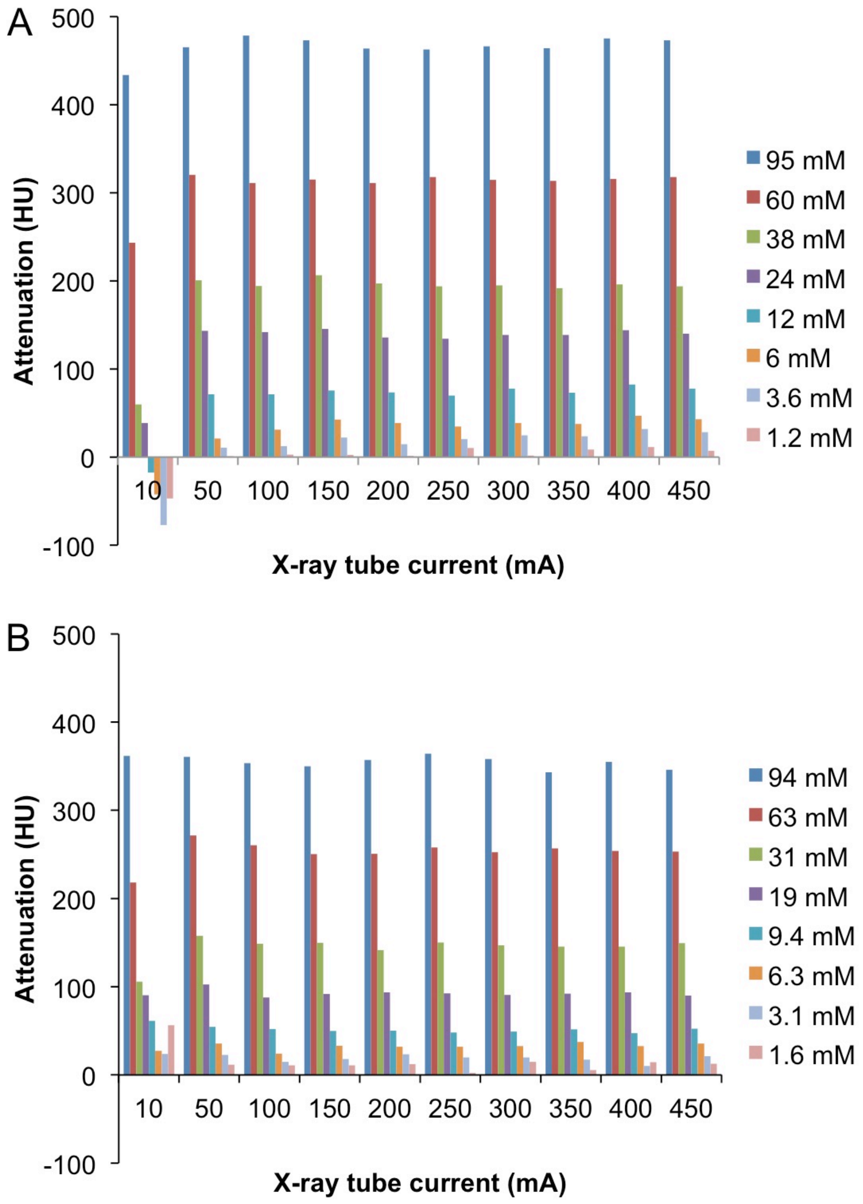


**Supplementary Figure 4** Attenuation of **A.** gold nanoparticle and **B.** iopamidol samples scanned at 80 kV and reconstructed with IMR at different X-ray tube currents.

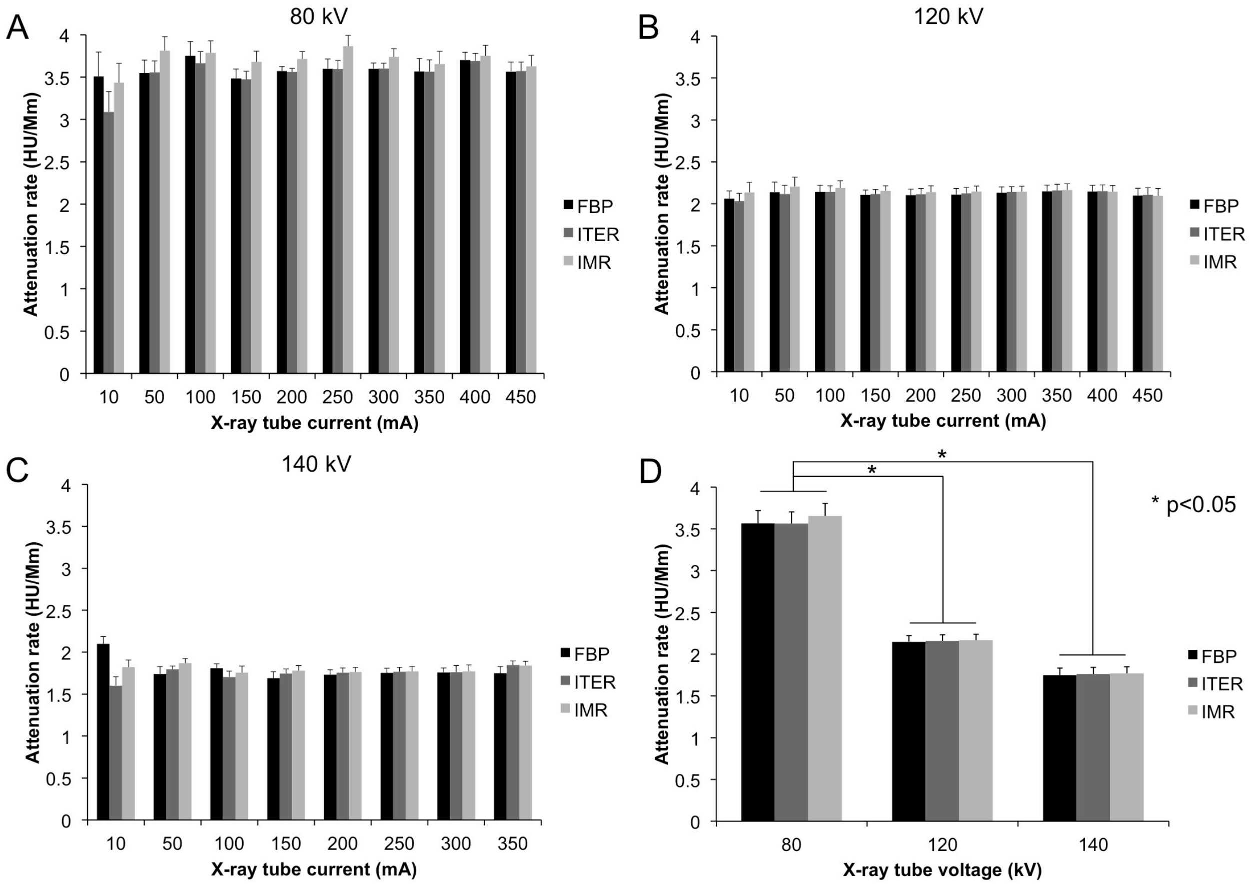


**Supplementary Figure 5 A-C.** Effect of x-ray tube current and reconstruction algorithm on the attenuation rate of iopamidol when scans are performed at 80, 120 and 140 kV. **D.** Comparison of the attenuation rate of iopamidol at different X-ray tube voltages when scans are performed at 350 mA.

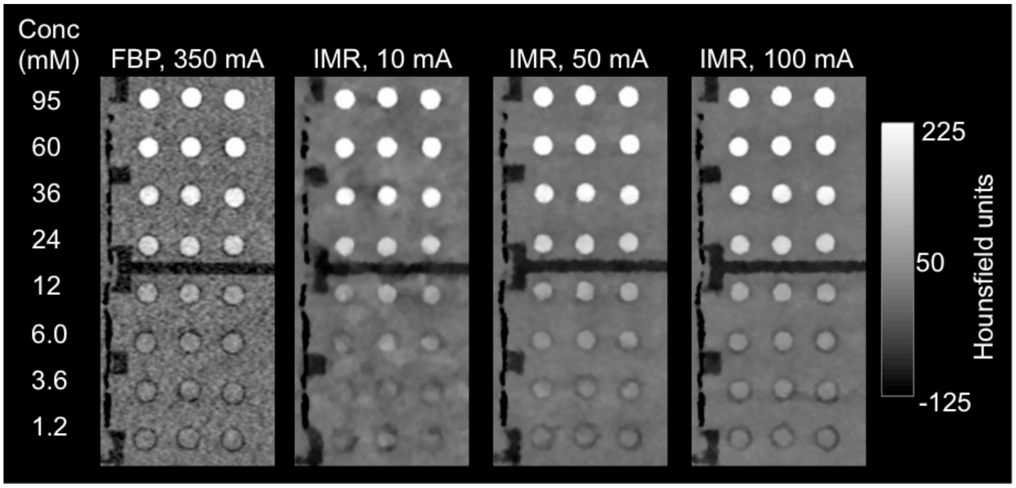


**Supplementary Figure 6** Comparison of images of the gold nanoparticle phantom acquired at high current and reconstructed with FBP, versus images acquired at low currents and reconstructed with IMR.

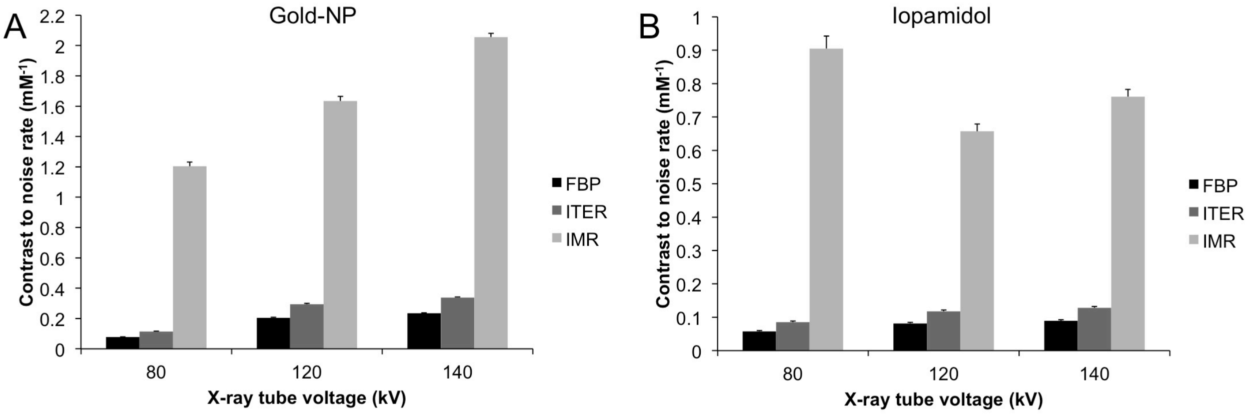


**Supplementary Figure 7** CNR rate for data acquired at 350 mA using different X-ray tube voltages and reconstruction algorithms for **A.** gold nanoparticles and **B.** iopamdiol.

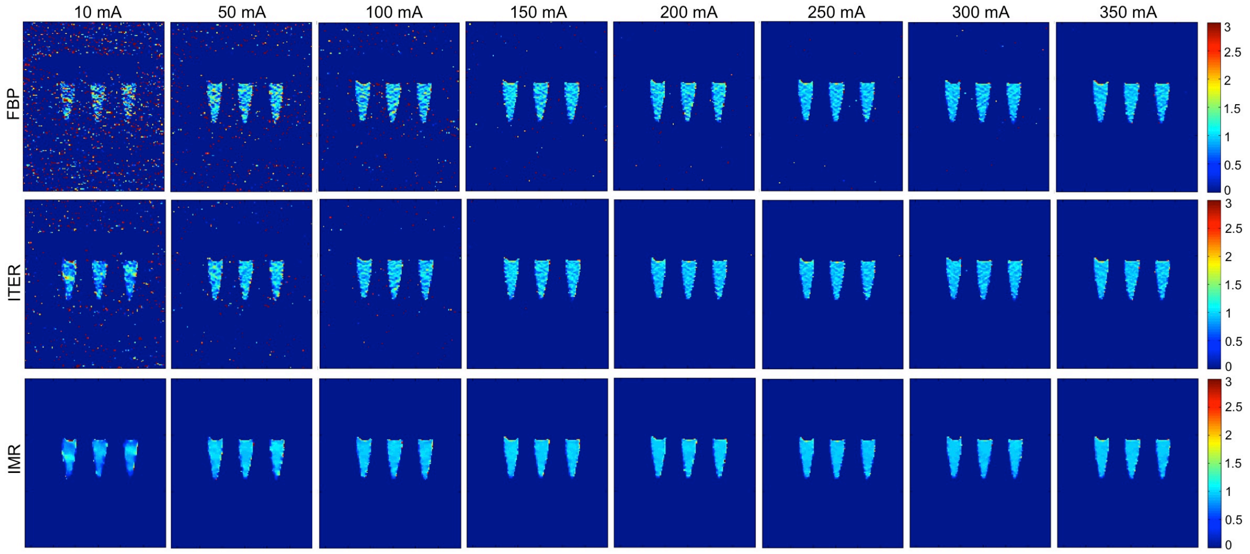


**Supplementary Figure 8** Dual energy ratiometric images formed from 80 kV and 140 kV images acquired at different currents and reconstructed using different algorithms.

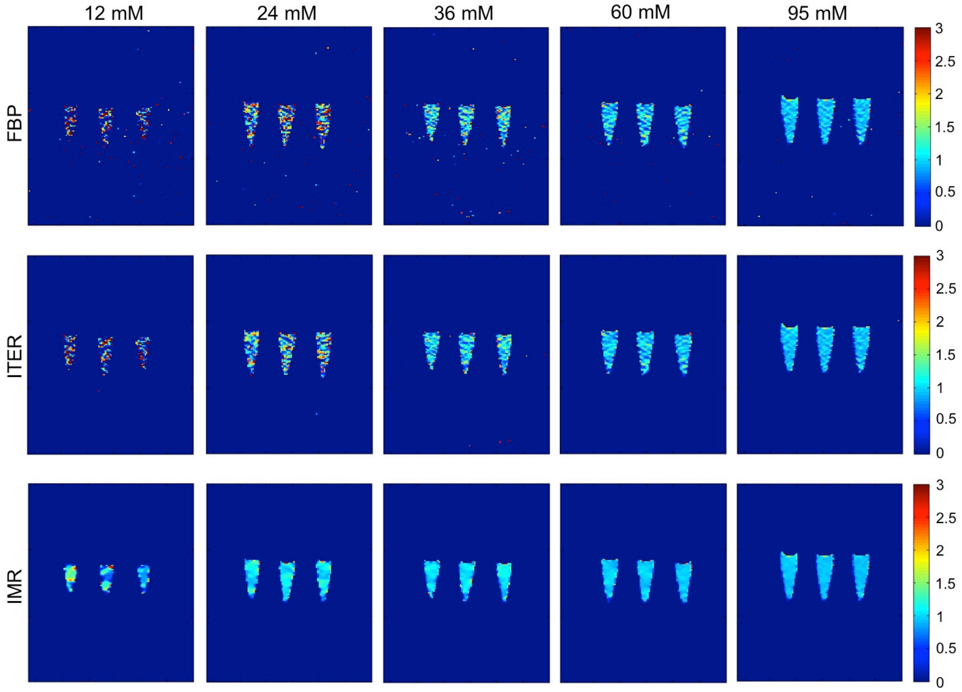


**Supplementary Figure 9** Dual energy CT ratiometric maps formed from 80 and 140 kV images acquired at 250 mA of different concentrations of contrast agents.

**Ratiometric map Matlab code.** Threshval corresponds to threshold value to be subtracted to all pixels of the original DICOM image. This operation aims at separating the object of concern from the background.

Note: The following code is written with notations as for images acquired using the combination of voltages of 80kV and 140kV. Analogue codes were used for other voltage combinations, adjusting the searching directory of the images and notations "I80", "I140", "thresh80", "thresh140" according to voltages, currents, reconstruction methods used.

```
I80 =
double(dicomread(strcat('C:\Users\Martina\Desktop\upenn_ct\GW06\FBP\I3910')))
;
I140 =
double(dicomread(strcat('C:\Users\Martina\Desktop\upenn_ct\GW26\FBP\I3910')))
;                 % Choice of the image using the corresponding directory

threshval=[1100]; % The threshold value was adapted for different studies


 %Initial images :
figure(3)
    imagesc(I80),
    axis image,
    title('I80 original-image'),
    colormap, colorbar;

figure(4)
    imagesc(I140),
    axis image,
    title('I140 original-image'),
    colormap, colorbar;


%Create the threshold values
for ThrVal = 1:length(threshval)

    thresh80 =  I80 - threshval(ThrVal);
    figure(5)
    imagesc(thresh80),
    axis image,
    title(strcat('I80 threshold-value:',  num2str(threshval(ThrVal)))),
    colormap , colorbar;

    thresh140 = I140 - threshval(ThrVal);
    figure(6)
    imagesc(thresh140),
    axis image,
```

```
    title(strcat('I140 threshold-value:', num2str(threshval(ThrVal)))),
    colormap , colorbar;

%Remove negative numbers

    for i = 1:512
        for j = 1:512

            if thresh80(i,j) < 0
               thresh80(i,j) = 0;
            end

            if thresh140(i,j) < 0
               thresh140(i,j) < 0;
            end


        end

    end


%Create the ratiometric maps

    ratio80_140 = thresh80./thresh140;

    ratio140_80 = thresh140./thresh80;


%Remove infinity and NaN values

    ratio80_140(isinf(ratio80_140))=0;

    ratio80_140 (isnan(ratio80_140))=0;

    ratio140_80(isinf(ratio140_80))=0;

    ratio140_80(isnan(ratio140_80))=0;


%Show the images
   figure(1)
        imagesc(ratio80_140),
        axis image,
        ylim([250 400]), xlim([175 300]),
        title(strcat('ratio80/140 threshold val:',
num2str(threshval(ThrVal)))),
        colormap , colorbar(gray), % colorbar(gray) to get a linear gray
                              scale used for standard deviation
                        determination
        caxis([0,3]);

    figure(2)
```

```
        imagesc(ratio140_80),
        axis image,
        ylim([250 400]), xlim([175 300]),
        title(strcat('ratio140/80 threshold-value:',
num2str(threshval(ThrVal)))),
        colormap , colorbar(gray),
        caxis([0,3]);

end
```

**Noise estimation.** Standard deviation of selected areas of interest was estimated with Matlab function as follows.

```
a=imread(strcat('C:\Users\Martina\Desktop\noise_analysis_images\all_voltages_
caxis0_1p3\GOLD\IRM_RATIO140_120_I3910NB.tif'));
figure(), imshow(a)

agray=rgb2gray(a)      %conversion of RGB image to grayscale image

[rows columns numberOfColorBands] = size(agray);
imtool(agray)                 %  Image  Viewer  displays  information  about  the
location.
```

Displayed image was cut using "crop" tool, exported to the Workspace "Ctrl+E" and standard deviation of the cropped image area (I) was computed using the function std2 for standard deviation calculation of an gray scale area: std2(I). For simplicity, calculations were performed on selected areas, conserving the same area for all examined images for each contrast agent (as the position of the probes was not the same for the two phantoms).
